# Tinea Nigra: Clinical and Diagnostic Guidance

**DOI:** 10.7759/cureus.66443

**Published:** 2024-08-08

**Authors:** Jesús Iván Martínez-Ortega, Ilse Fernández-Reyna, Carlos Enrique Atoche Dieguez, Lourdes Espinosa Alonzo, Arely Gissell Ramirez Cibrian

**Affiliations:** 1 Dermatology, Dermatological Institute of Jalisco, Zapopan, MEX; 2 Mycology, Dermatological Center of Yucatan, Mérida, MEX; 3 Medical Benefits, Mexican Social Security Institute, Campeche, MEX

**Keywords:** pigmented macules, superficial fungal infection, mycological diagnosis, tropical infections, tropical mycoses, palmar lesion, fungal infection, hortaea werneckii, phaeohyphomycosis, tinea nigra

## Abstract

Tinea nigra is a rare superficial fungal infection characterized by asymptomatic, unilateral, well-defined brown to black macules predominantly affecting the palms and soles. Diagnosis is often challenging due to its rarity and resemblance to other pigmented lesions. This report presents a clinical case, a diagnostic algorithm, and treatment recommendations, emphasizing the role of thorough examination and questioning.

We describe the case of a 64-year-old woman of Amerindian (Maya) heritage from Yucatan, Mexico, who presented with a three-month history of a slowly growing dark spot on her left palm. The lesion was asymptomatic, non-scaling, and non-palpable. Palmar skin scrapings, prepared with KOH, revealed pigmented yeast and hyphae, leading to a diagnosis of tinea nigra. Following treatment with topical ketoconazole, the patient's lesions completely resolved at the one-month follow-up. The cultivation of scales confirmed the presence of *Hortaea werneckii*.

Our findings highlight the importance of considering tinea nigra in the differential diagnosis of pigmented lesions on acral surfaces. We propose a diagnostic algorithm to aid healthcare professionals in recognizing this uncommon condition and recommend treatment protocols that effectively resolve the infection within two weeks. This case underscores the necessity for increased awareness and accurate diagnosis of tinea nigra, particularly in non-endemic regions.

## Introduction

Tinea nigra, a rare superficial phaeohyphomycosis, manifests as asymptomatic, unilateral, well-defined brown to black macules typically affecting the palms or soles [1,2]. It is caused by the dematiaceous fungus* Hortaea werneckii*, previously classified under *Exophiala*, *Phaeoannellomyces*, and *Cladosporium *[3,4]. Other fungal genera can also rarely cause this infection, such as *Stenella araguata* and *Curvularia lunata*, with these being the only reported cases of tinea nigra caused by fungi other than *Hortaea werneckii* [3,4]. First identified in 1891 by Alexandre Cerqueira in Salvador (Bahia), Brazil, it was initially termed keratomycosis nigra palmaris [2].

Clinically, tinea nigra presents as pigmented macular patches on the palms or soles, with simultaneous involvement of both sites being uncommon. It predominantly occurs in tropical and subtropical regions, though isolated cases have been reported globally among travelers from endemic areas. The incubation period typically ranges from several weeks to 18 months based on a retrospective study [1]. Although an experimental study suggested the potential for infection to manifest up to 20 years later, this has not been clinically proven [2].

Due to its epidemiological rarity, tinea nigra is often not suspected upon encounter. A retrospective study found that dermatologists correctly diagnosed the condition in only 44% of cases, while general practitioners did so in just 2.6% [2]. For accurate diagnosis, dermoscopy provides key visual features essential for differentiation, while laboratory confirmation can be achieved through microscopic examination of KOH preparations, which reveals characteristic fungal morphology [2]. Although not necessary for confirmation, cultures using media such as Sabouraud dextrose agar and incubating at 25-30°C can be used for further identification if needed [1]. Here, we present a clinical case of tinea nigra, offer clinical recommendations, and propose three diagnostic algorithms to aid in recognizing and diagnosing this uncommon condition. The first algorithm is designed for healthcare professionals without access to dermoscopy, guiding them through initial assessment and diagnosis. The second algorithm is tailored for dermatologists using dermoscopy, highlighting key dermoscopic features crucial for accurate identification. The third algorithm focuses on mycological aspects, providing guidance on laboratory confirmation through microscopic examination of KOH preparations and culture techniques.

## Case presentation

**Figure 1:**
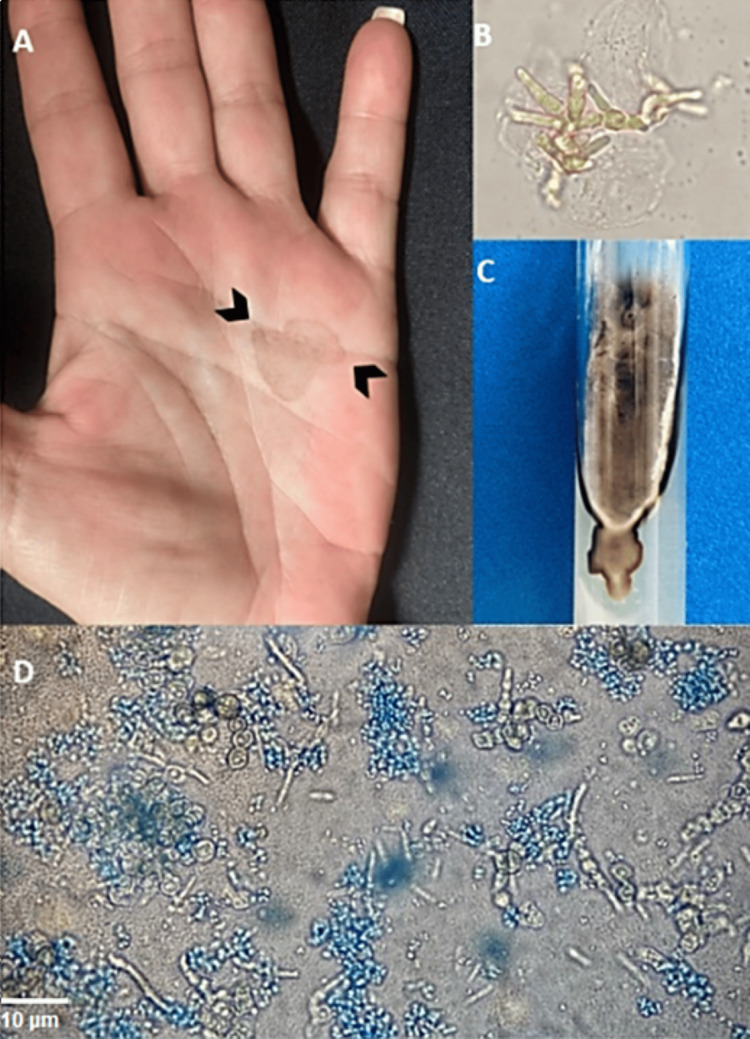
The diagnostic process for tinea nigra Panel A shows the clinical lesion on the palm. Panel B presents the KOH 20% preparation revealing pigmented yeast and hyphae. Panel C depicts the culture, with black-colored cottony colonies after 30 days. Panel D highlights the morphological aspects of the subculture, showing oval, dark, and hyaline yeast-like cells along with dark-colored, tabulated hyphae (magnification: 400x). The dye used in Panel D is lactophenol cotton blue.

A 64-year-old woman of Amerindian (Maya) heritage, living in Yucatan, Mexico, presented to the dermatology clinic with a three-month history of a slowly growing dark spot on her left palm. The lesion was asymptomatic, and the patient reported no use of citrus products or recent travel. Upon examination, a non-scaling, non-palpable brown patch on the left palm was noted (Figure 1) (Panel A). Even after cleaning the lesion with soap and water, it persisted. Palmar skin scrapings, prepared with KOH, revealed pigmented yeast and hyphae (Panel B), leading to a diagnosis of tinea nigra. Following treatment with topical ketoconazole, the patient's lesions completely resolved at the one-month follow-up. Cultivation of scales confirmed the presence of *Hortae werneckii*, with the isolation of cottony colonies exhibiting a black color (Panel C) after 30 days. Oval, dark, and hyaline yeast-like cells, along with dark-colored, tabulated hyphae, were observed (Panel D).

## Discussion

As previously mentioned, tinea nigra is exceptionally rare, occurring in only 0.085% of recorded mycoses. Indeed, we found only three studies documenting 12, 22, and 50 cases [1-3]. This condition primarily affects the palms in over 50% of cases [1-3]. Although it predominantly occurs in tropical and subtropical regions, it can also appear in travelers returning from these areas [1].

Due to its rarity, the pathogenesis of tinea nigra has not been extensively studied. However, it has been shown that the causative organism lacks virulence factors, cannot grow at 37°C, does not produce siderophores, and most strains are unable to alter the pH of their microenvironment. These characteristics suggest a low pathogenicity for this species, indicating it may be more of a colonization than an infection [2,5,6]. Nonetheless, there are two presumptive reports of *Hortaea werneckii *causing infections in peritonitis and splenic abscess, behaving as an opportunistic pathogen in these cases [7,8]. Interestingly, no keratinolysis is observed, and adhesion to acral surfaces is believed to be due to the hydrophobic nature of the yeast cells [2].

We hypothesize that, aside from the van der Waals forces between the lipidic components of the fungal cell walls, the hydrophobic effect, which is based on the second law of thermodynamics stating that entropy in a system tends to increase, might play a significant role in the presence of an aqueous salinized milieu [9]. This hypothesis could explain some of the clinical features observed. The soles and palms present the thickest stratum corneum. The granular layer beneath, along with some cells of the spinous layer, produce lipids that form a barrier in the form of lamellar bodies, which are secreted by exocytosis into the extracellular space [10].

The hydrophobic lipid matrix of the stratum corneum creates an environment where hydrophobic interactions are favored, facilitating the aggregation and stability of hydrophobic fungi. This aggregation minimizes their exposure to water, aligns with the principles of the hydrophobic effect, and allows them to thrive and attach together in the hydrophobic environment of the stratum corneum. Clinically, this may explain why they are seen as a single circular macule, as multiple macules would not be thermodynamically favored. Additionally, the presence of saline water, which has a higher polarity, may further promote these hydrophobic fungi to clump together, enhancing their attachment to the stratum corneum [9]. Finally, the clinical pigmentation is caused by the presence of the fungus itself. The chromophore 1,8-dihydroxy naphthalene (DHN)-melanin is synthesized and deposited in granules on the outer side of the cell wall. Therefore, it can be assumed that the intensity of the pigmentation and the size of the clinical macule correlate with the quantity of microorganisms present [6].

**Figure 2:**
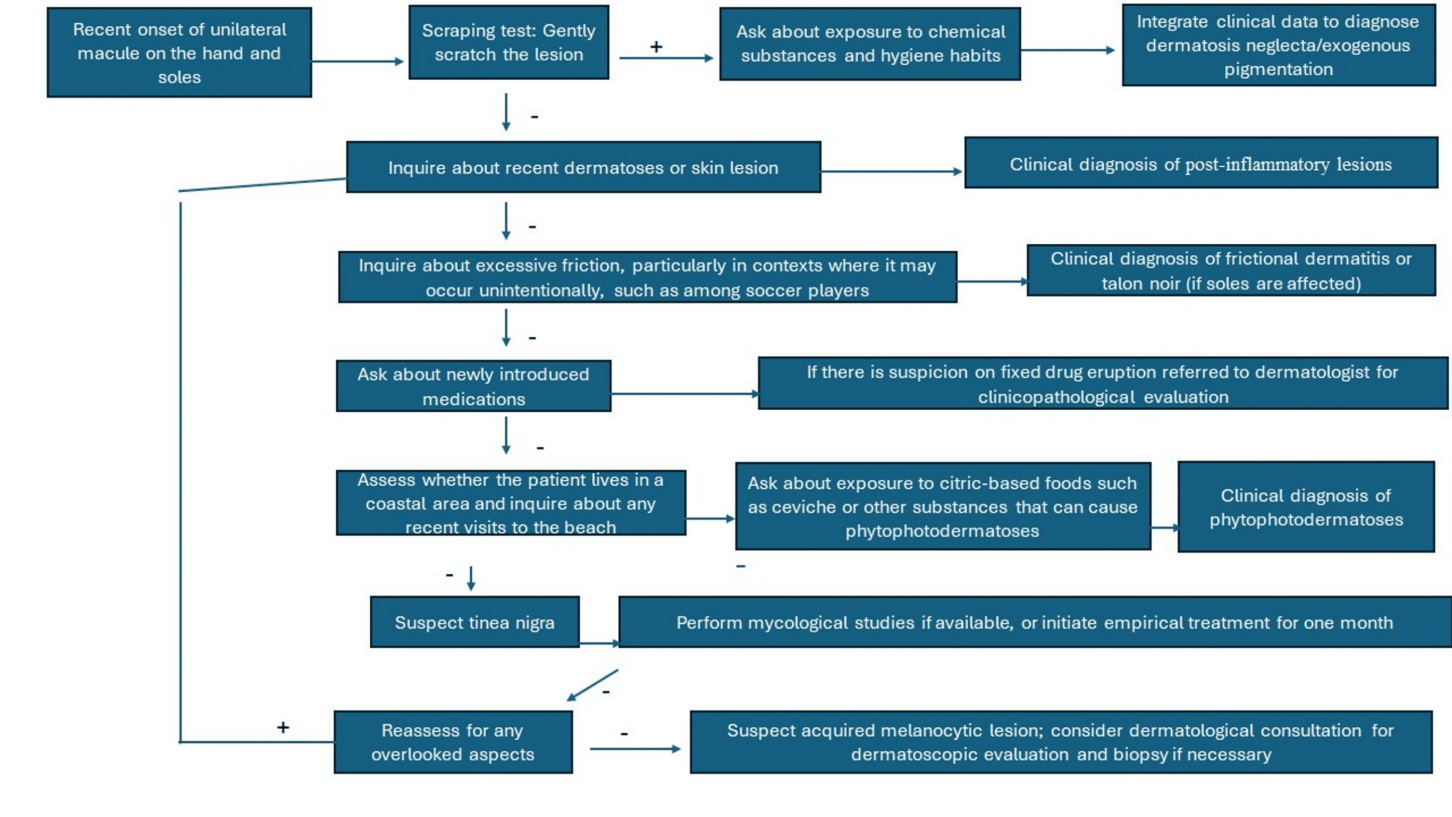
Simplified diagnostic algorithm for tinea nigra for general practitioners The scheme presents a simplified diagnostic algorithm for tinea nigra, designed for general practitioners, family physicians, and clinicians providing initial care. It highlights that an effective clinical diagnosis can be achieved through comprehensive examination and patient questioning. Image credits to Jesus Martinez, MD.

Differential diagnosis and a diagnostic algorithm are depicted in Figure 2, focusing on general practitioners, family physicians, and clinicians providing initial medical attention. Clinical diagnosis can be effectively achieved through thorough examination and questioning. The image primarily reflects cases with early onset and involvement of the soles and palms. Chronic lesions or those affecting the fingers or exhibiting bilateral involvement may require a different diagnostic approach. While instances following experimental inoculation have been documented up to 20 years earlier, these are uncommon [11]. Additionally, macules affecting the fingers and bilateral involvement have been reported in cases of tinea nigra, but they are exceptional [1]. Therefore, the diagnostic workflow for such cases should be more of an exclusion process and differ from the one presented.

**Figure 3:**
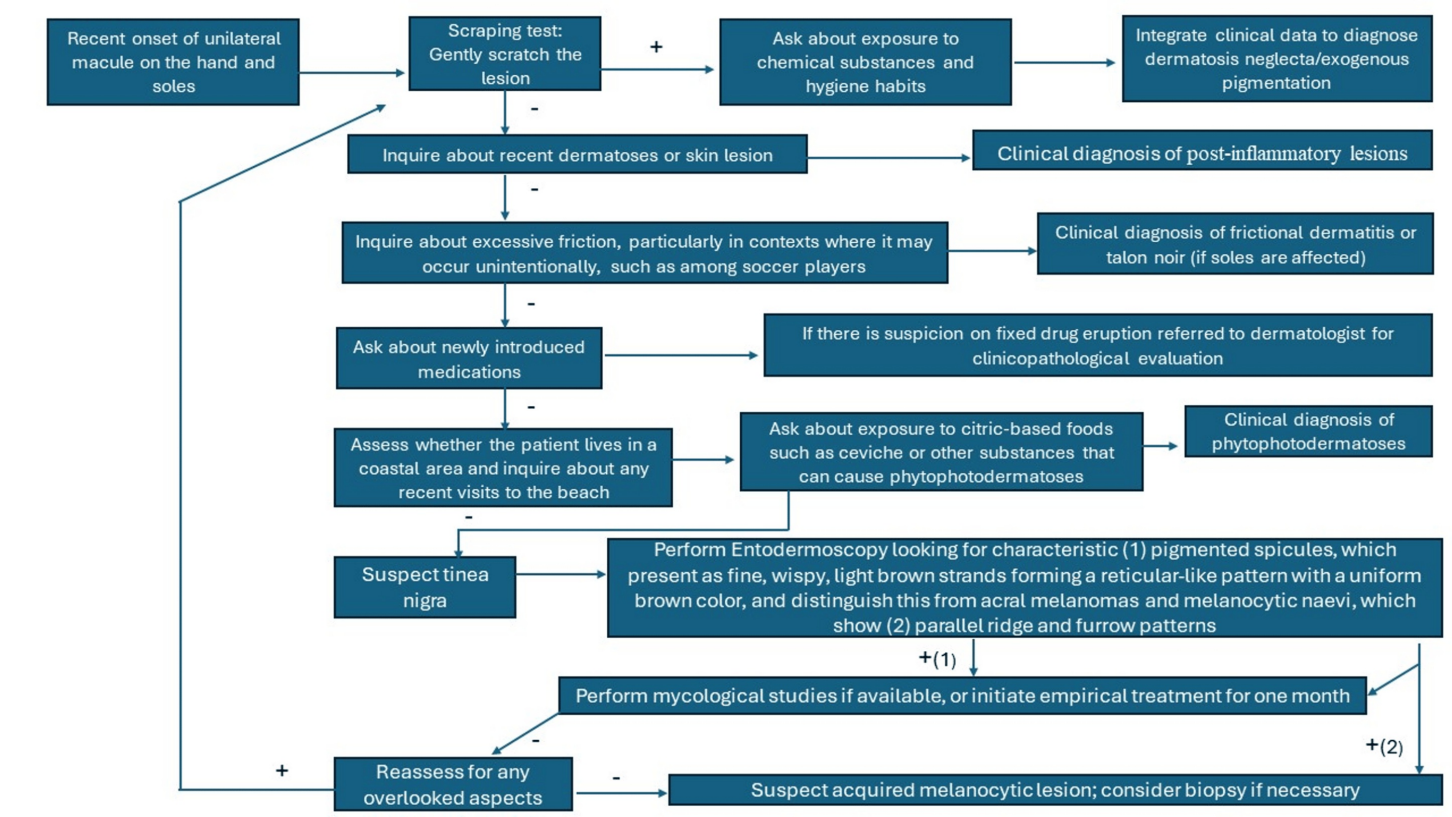
Simplified dermatoscopic diagnostic algorithm for tinea nigra This figure is designed for dermatologists and physicians with dermatoscopic experience, illustrating the key dermoscopic features of tinea nigra as reported in the literature to aid in accurate diagnosis. Note that (1) highlights the dermoscopic features characteristic of tinea nigra, while (2) shows the dermoscopic features of melanocytic lesions. Depending on the findings, the dermatologist may choose to confirm with mycological tests if there is uncertainty or proceed directly with a biopsy. Image credits to Jesus Martinez, MD.

**Figure 4:**
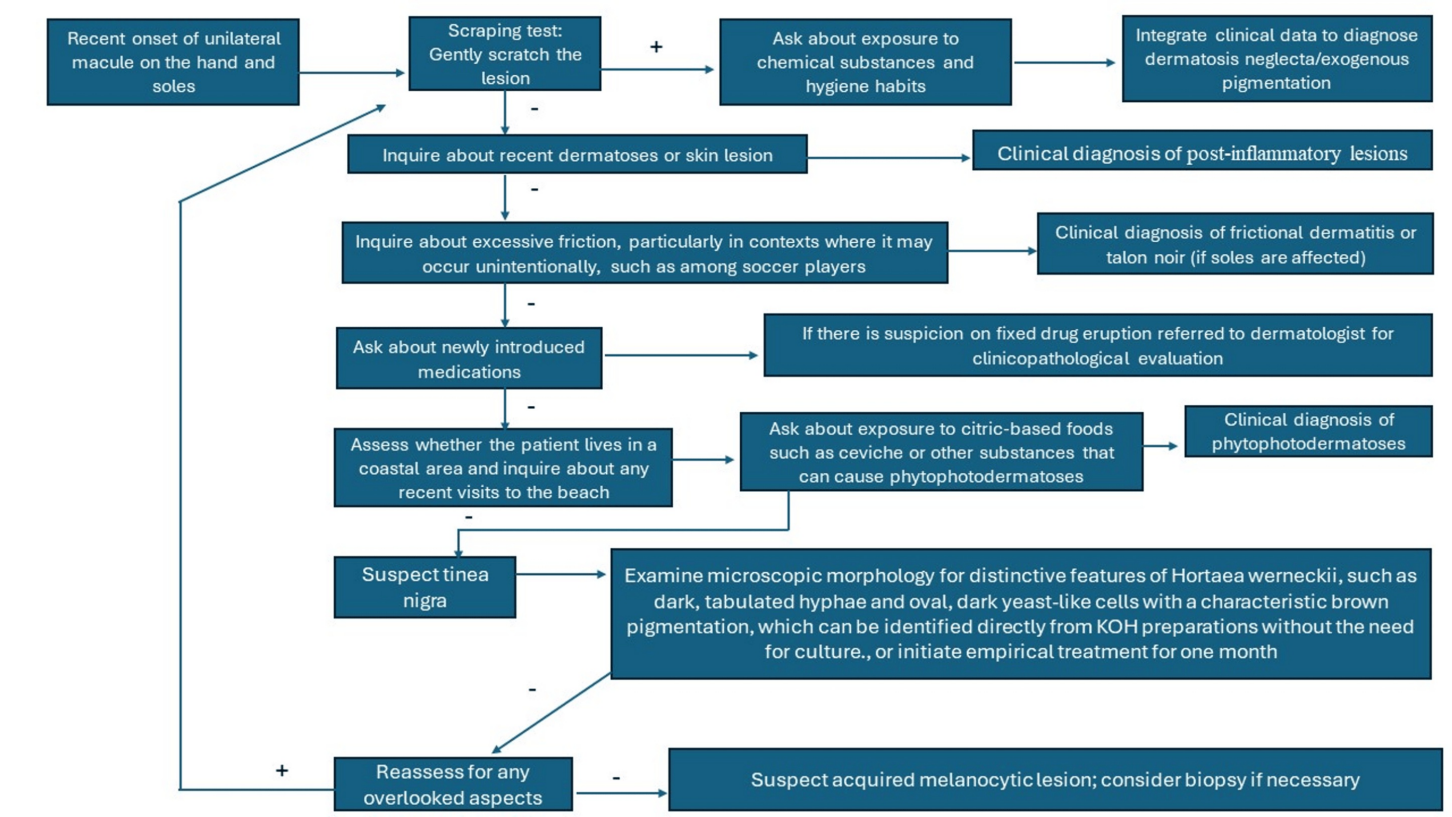
Simplified mycological diagnostic algorithm for tinea nigra Intended for dermatologists equipped with microscopes and KOH preparations or mycologists, this figure highlights the distinctive mycological morphology of *Hortaea werneckii*, emphasizing features that are identifiable even without culture. Image credits to Jesus Martinez, MD.

Rare diagnoses were not discussed in this initial encounter approach. However, other rarer etiologies such as atypical syphilis, pinta, or pityriasis versicolor should not be discarded in cases with atypical progression or evolution. Dermoscopy was not included in the initial assessment as it is not commonly used by non-dermatologists, but it is recommended when available due to its increasing utility [1-3]. Piliouras et al. found the overall sensitivity of clinical diagnosis for tinea nigra to be notably low at 14.0% [2]. Dermoscopy significantly enhanced diagnostic accuracy, correctly identifying tinea nigra in 53.8% (seven out of 13) of cases where it was used, compared to only 2.7% (one out of 37) when dermoscopy was not utilized. For detailed dermatoscopic features relevant to diagnosing tinea nigra, including characteristic patterns and their interpretation, refer to Figure 3 [2]. This figure is designed for use by dermatologists and any physicians with dermatoscopic training. For specific mycological characteristics that can be identified without culture, such as unique KOH preparation features of *Hortaea werneckii*, see Figure 4 [1]. This figure is intended for dermatologists with microscopy expertise or mycologists. Recently, the first clinical applications of confocal microscopy have been reported, and as more clinical evidence accumulates, it may become an integral part of the diagnostic algorithm in the future [12].

Treating tinea nigra typically leads to resolution within two weeks when using keratolytic agents such as urea, salicylic acid, and Whitfield ointment (comprising salicylic acid 3%, benzoic acid 2-6% in a suitable base), or through the application of topical antifungal medications once or twice daily [1-3]. 

Finally, the main limitation of this case report is the absence of dermoscopic images of tinea nigra. Given the descriptive nature of the report, including these images would have complemented the diagnostic algorithms presented and provided useful comparison and illustration.

## Conclusions

Tinea nigra, while rare, is diagnosable with appropriate clinical assessment. This study emphasizes the critical role of detailed history-taking and thorough examination for general practitioners in diagnosing this uncommon condition. For accurate diagnosis and confirmation, Figure 2 outlines a simplified diagnostic algorithm tailored for general practitioners, while Figure 3 presents essential dermoscopic features for dermatologists and physicians with dermatoscopic experience. Figure 4 details the distinctive mycological morphology of *Hortaea werneckii *observed in KOH preparations, which aids in confirming the diagnosis. Effective treatment, typically involving keratolytic agents or topical antifungals, usually resolves the condition within two weeks. The recent introduction of confocal microscopy holds promise for enhancing diagnostic precision and utility in the future as more clinical evidence becomes available.

## References

[1] Bonifaz A, Badali H, de Hoog GS (2008). Tinea nigra by Hortaea werneckii, a report of 22 cases from Mexico. Stud Mycol.

[2] Piliouras P, Allison S, Rosendahl C, Buettner PG, Weedon D (2011). Dermoscopy improves diagnosis of tinea nigra: a study of 50 cases. Australas J Dermatol.

[3] Perez C, Colella MT, Olaizola C, Hartung de Capriles C, Magaldi S, Mata-Essayag S (2005). Tinea nigra: report of twelve cases in Venezuela. Mycopathologia.

[4] Al-Odaini N, Wei JY, Zheng YQ, Zheng DY, Khader JA, Cao CW (2022). A special tinea nigra caused by Curvularia lunata: case report and literature review. Mycopathologia.

[5] de Hoog GS, Gerrits van den Ende AH (1992). Nutritional pattern and eco-physiology of Hortaea werneckii, agent of human tinea nigra. Antonie Van Leeuwenhoek.

[6] Plemenitas A, Vaupotic T, Lenassi M, Kogej T, Gunde-Cimerman N (2008). Adaptation of extremely halotolerant black yeast Hortaea werneckii to increased osmolarity: a molecular perspective at a glance. Stud Mycol.

[7] Chamroensakchai T, Kleebchaiyaphum C, Tatiyanupanwong S, Eiam-Ong S, Kanjanabuch T (2021). Tinea nigra palmaris-associated peritonitis, caused by Hortaea werneckii: the first case report in a peritoneal dialysis patient. Perit Dial Int.

[8] Ng KP, Soo-Hoo TS, Na SL (2005). The mycological and molecular study of Hortaea werneckii isolated from blood and splenic abscess. Mycopathologia.

[9] Suvarna KS, Layton C, Bancroft JD (2018). Bancroft's theory and practice of histological techniques E-Book. https://books.google.com.mx/books/about/Bancroft_s_Theory_and_Practice_of_Histol.html?id=wPPU4NyGm3gC&redir_esc=y.

[10] Ross MH, Pawlina W (2017). Correlación con biología molecular y celular. Ross, Histology: A Text and Atlas.

[11] Blank H (1979). Tinea nigra: a twenty-year incubation period?. J Am Acad Dermatol.

[12] Veasey JV, Avila RB, Ferreira MA, Lazzarini R (2017). Reflectance confocal microscopy of tinea nigra: comparing images with dermoscopy and mycological examination results. An Bras Dermatol.

